# Anomalous Innervation to the Extensor Digitorum Brevis

**DOI:** 10.1055/s-0039-1685531

**Published:** 2019-05-02

**Authors:** Marc A. Swerdloff, Danielle F. Stewart

**Affiliations:** 1 Department of Neurology, Marcus Neuroscience Institute, Boca Raton Regional Hospital, Boca Raton, Florida, United States; 2 Clinical Affiliate Faculty Department of Neurology at Florida Atlantic University, Boca Raton, Florida, United States; 3 Department of Neurology, Nova Southeastern College of Osteopathic Medicine, Fort Lauderdale, Florida, United States

The nerve supply of the extensor digitorum brevis (EDB) usually arises from the deep peroneal nerve. An anomalous innervation arising from the accessory deep peroneal nerve is described in 15% of cases. 1 An “all tibial foot” innervation pattern has been reported. 2 3

In our clinic, electromyographic findings revealed the presence of a deep peroneal to posterior tibial nerve anastomosis similar to the forearm anastomosis of the median to ulnar nerve of Martin and Gruber.

**Fig. 1:**
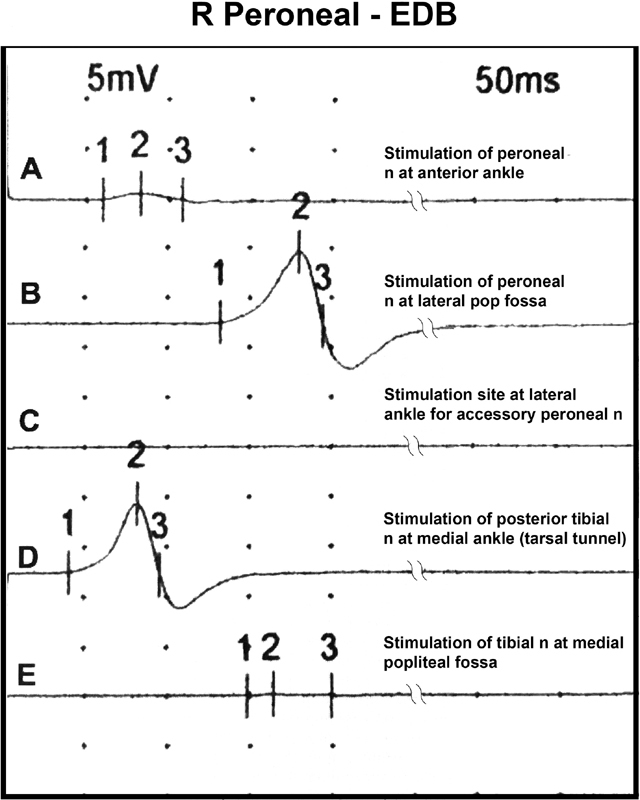
Compound muscle action potential (CMAP) of the extensor digitorum brevis (EDB) stimulating from various points on the foreleg. Normally the amplitude of the CMAP of the EDB obtained by stimulation of the deep peroneal nerve at the ankle would be equal to that obtained by stimulation at the popliteal fossa, that is, trace A would equal with B. With a deep peroneal to posterior tibial anastomosis, a response is elicited by stimulating the posttibial nerve at the medial ankle (trace D). If there was an accessory deep peroneal nerve contribution, trace C would have a response. Proximal tibial stimulation would elicit a robust response in trace E in an “all tibial foot.”

**Fig. 2:**
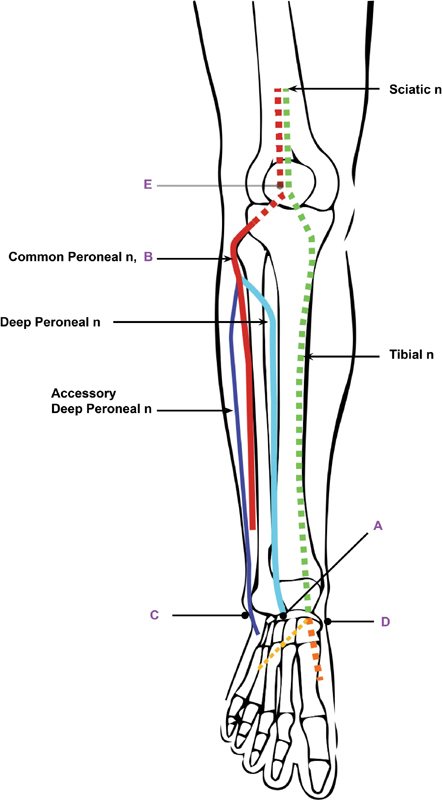
Stimulation sites used to obtain a compound muscle action potential (CMAP) of the extensor digitorum brevis (EDB). ( **A** ) anterior ankle, ( **B** ) Lateral popliteal fossa, ( **C** ) lateral ankle, ( **D** ) medial ankle, tarsal tunnel, and ( **E** ) medial popliteal fossa.

**Fig. 3:**
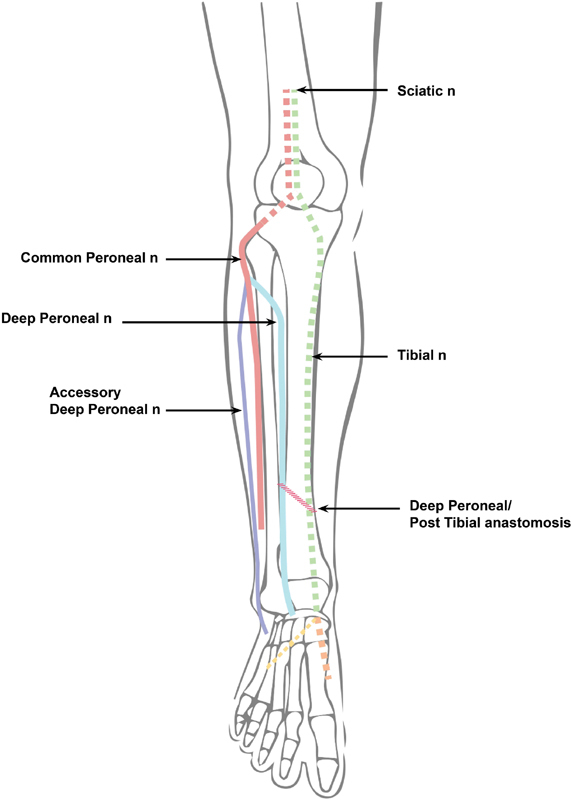
Illustration of deep peroneal/post tibial anastomosis that must be present to obtain our results. It is shown as red slanted lines.

In Fig. 1 , tracings of the EDB compound muscle action potential (CMAP) were generated after stimulation at various sites in the foreleg ( Fig. 2 ). The medially located tarsal tunnel is a site that will not generate an EDB CMAP unless there is an anomalous innervation to the EDB. In our cases, the distal peroneal site of stimulation failed to generate a maximal CMAP. Additional amplitude occurred after stimulation of the distal tibial nerve in the tarsal tunnel. Proximal peroneal and distal tibial nerve stimulations are characteristics of a cross over in the foreleg from the peroneal to the tibial nerve ( Fig. 3 ).

Of 72 patients, 11% (8/72) showed these results. It was present in both legs in half of the patients (4/8).

The finding of a deep peroneal to posterior tibial anastomosis will be of interest to neurologists performing electromyographic studies and surgeons that deal with injuries to the foreleg.

In our sample, it was more frequent than the more widely known accessory deep peroneal variant.
